# Fluconazole plus flucytosine is a good alternative therapy for non‐HIV and non‐transplant‐associated cryptococcal meningitis: A retrospective cohort study

**DOI:** 10.1111/myc.12944

**Published:** 2019-06-09

**Authors:** Zhanyi Li, Yu Liu, Yutian Chong, Xiangyong Li, Yusheng Jie, Xiaoyan Zheng, Ying Yan

**Affiliations:** ^1^ Department of Infectious Diseases Third Affiliated Hospital of Sun Yat‐sen University Guangzhou China; ^2^ Department of General Surgery (Thyroid and Breast) Third Affiliated Hospital of Sun Yat‐sen University Guangzhou China

**Keywords:** amphotericin B (AmB), cryptococcal meningitis (CM), fluconazole (FCZ)

## Abstract

Cryptococcal meningitis (CM) carries a high risk of mortality with increasing incidences in immune competent hosts. Current treatments are not well tolerated, and evaluation of other treatments is needed. Fluconazole and 5‐flucytosine in treating immune competent hosts have not been characterised. To evaluate the efficacy of fluconazole and 5‐flucytosine in treating non‐HIV‐ and non‐transplant‐associated CM. We performed a retrospective cohort study of the outcomes in immune competent patients with CM treated with fluconazole and 5‐flucytosine or deoxycholate‐amphotericin B and 5‐flucytosine. The primary outcome was treatment response evaluated at the 12th week after initiation of antifungal therapy. A total of 43 and 47 patients received amphotericin B deoxycholate and 5‐flucytosine or fluconazole and 5‐flucytosine, respectively. A total of 38 (88.4%) patients cannot tolerate recommended doses of amphotericin B deoxycholate and 5‐flucytosine (patients needed dose reduction during the treatment). Patients given fluconazole and 5‐flucytosine had higher baseline cryptococcal burdens (median 3632 versus 900 cryptococci/mL, *P* = 0.008). No significant differences were seen in cryptococcus clearance (74.4% vs 70.2%, *P* = 0.814), treatment time (39 days, 20‐69 days vs 21 days, 7‐63 days, *P* = 0.107) and successful response (including complete and partial responses) rates (69.7% vs 72.3%, *P* = 0.820). Fluconazole and 5‐flucytosine treatment had lower total adverse events (19.1% vs 90.7%, *P* < 0.001). Fluconazole and 5‐flucytosine had relatively high efficacy with few adverse events in treating CM. Fluconazole and 5‐flucytosine therapy is promising in patients that do not tolerate or are not suited for amphotericin B deoxycholate treatment.

## 1 INTRODUCTION

*Cryptococcus neoformans* (*C. neoformans s.l*.) and *Cryptococcus gattii* (*C. gattii s.l*.) species complex are opportunistic pathogens, and cryptococcal meningitis (CM) is the most common cause of fungal meningitis by a considerable high morbidity and mortality. It was estimated the global incidence of CM to be substantial at 223 100 cases annually, resulting in 181 100 annual deaths in 2014.^1^ This infection is common in immunosuppressed patients including those with HIV or who have undergone solid organ transplantation.^2^, ^3^, ^4^ There has been an increase in the incidence of this disease among immunocompetent hosts that are not HIV‐infected or not transplant recipients in recent years.^5^, ^6^, ^7^ Reports have noted that about half of all reported cases of cryptococcosis are in non‐HIV‐infected and non‐transplant patients (between 44% and 55%).^8^, ^9^, ^10^, ^11^ Additionally, CM is associated with excessively high disability and mortality rates.^12^, ^13^ Induction therapy is a critical treatment for CM in non‐HIV‐infected and non‐transplant CM patients. Induction therapy of amphotericin B (AmB) (0.7‐1.0 mg/kg per day) combined with 5‐flucytosine (5‐FC) (100 mg/kg per day) for at least four weeks is the globally preferred regimen for non‐HIV‐infected and non‐transplant patients with CM worldwide.^14^ However, high doses of AmB and 5‐FC can result in severe toxic side effects such as phlebitis, liver impairment, renal impairment, haematological impairment, myocardial damage and a majority of CM patients do not tolerate the recommended dosages.^15^, ^16^, ^17^, ^18^, ^19^, ^20^ The triazole antifungal drug fluconazole (FCZ) has been widely used in treating deep mycosis and has low rates of adverse events. FCZ combined with 5‐FC was recommended to treat HIV‐associated CM.^14^ However, FCZ combined with 5‐FC in treating non‐HIV‐ and non‐transplant‐associated CM has not been well characterised and thus the data are limited. Thus, in this study we compared the efficacy of combination therapies using AmB and 5‐FC versus FCZ and 5‐FC. Our goals were to analyse this drug combination therapy in order to propose alternative therapeutic regimens for CM in patients without HIV or patients that are not transplant recipients.

## 2 PATIENTS AND METHODS

### 2.1 Patients and definitions

We performed a retrospective review of all patients diagnosed with non‐HIV‐ and non‐transplant‐associated CM at the Third Affiliated Hospital of Sun Yat‐sen University between January 2010 and December 2017. The diagnosis was identified according to physical signs and symptoms along with at least one of the following factors: positive India ink stain microscopy of the CSF or a positive CSF culture for *Cryptococcus*.^21^ The patients were confirmed to be HIV‐negative based on negative results of serum HIV antibody tests, and medical records were examined to ensure no patient had undergone organ transplantation. These patients were divided into two groups according to treatment regimens they have received. In group I, the patients were treated with a combination of AmB and 5‐FC. In group II, the patients were treated with a combination of FCZ and 5‐FC. Exclusion criteria included (a) presence of severe hepatic or renal damage; (b) antifungal therapy administration before admission; (c) prior surgical intervention due to intracranial hypertension; and (d) recurrent CM.

This study was approved by the Ethics Committees of The Third Affiliated Hospital of Sun Yat‐Sen University. The study was in compliance with the Declaration of Helsinki and its later amendments. All study participants provided informed consent. Identifiable data involving the individuals in this study were encrypted.

### 2.2 Laboratory examination

Enrolled patients underwent lumbar punctures (LP) at least once a week in accordance with treatment guidelines.^14^, ^22^ Then CSF open pressure, CSF white blood cell count and classification, glucose, protein, India ink stain and cultures were recorded. In addition, other conventional blood tests and imaging studies including brain computerised tomography (CT) and/or magnetic resonance imaging (MRI) were also performed during treatment. CSF burden of cryptococcal organisms was evaluated by CSF cryptococcal organisms count through India ink stain. We use canavanine‐glycine‐bromothymol (CGB) medium to distinguish *C. gattii s.l*. from *C. neoformans s.l*. CGB medium showed clear colour change for *C. gattii s.l*. and can be used to differentiate it from *C. neoformans s.l*.^23^

### 2.3 Therapeutic methods

In group I, all patients were treated with amphotericin B deoxycholate (AmB): 0.7‐1.0 mg/kg per day and 5‐flucytosine (5‐FC):100 mg/kg per day.^14^ In group II, all patients were treated with fluconazole (FCZ): 400‐800 mg/d and 5‐flucytosine (5‐FC):100 mg/kg per day.^14^ During the course of treatment in the AmB and 5‐FC group, patients treated with intravenous AmB actually received an average maximum tolerated dosage of approximately 0.57 ± 0.98 mg/kg (range 0.3521‐0.7778 mg/kg) daily plus oral 5‐FC at an average maximum tolerated dosage of 57.75 ± 14.30 mg/kg (range 37.50‐78.95 mg/kg) daily. In FCZ and 5‐FC group, the patients were treated with oral 5‐FC at an average maximum tolerated dosage of 57.64 ± 13.34 mg/kg (range 38.46‐88.89 mg/kg) daily.

### 2.4 Outcome assessments

The primary outcome was treatment response evaluated at the 12th week after initiation of antifungal therapy. Therapeutic outcomes were classified into five levels: (a) complete response: survival and resolution of all attributable symptoms and signs of disease with CSF clearance; (b) partial response: survival and CSF clearance with persistence of attributable symptoms and signs of disease; (c) successful response rate: including both complete and partial responses; (d) stable response: survival with minor or no improvement in attributable symptoms and signs of disease and persistently positive CSF culture results; (e) disease progression: worsening clinical disease symptoms or signs and persistently positive CSF culture results; and (f) death: death during the prespecified evaluation period, regardless of cause.^24^ CSF clearance means negative of CSF cryptococcal organisms culture and CSF cryptococcal organisms count through India ink stain.

### 2.5 Assessment of adverse events

Data of adverse events were collected and assessed by National Cancer Institute Common Terminology Criteria for Adverse Events version 5.0 (NCI CTCAE v5.0).

### 2.6 Statistical analysis

Baseline demographic and clinical characteristics are presented as percentages, mean with standard deviations (SD) or medians with range; comparisons were performed using the chi‐square or Fisher's exact tests for categorical data, and with Student's t or Mann‐Whitney U tests for continuous data. Efficacy of treatment responses was estimated using the Wilcoxon rank sum test based on the five grades over ten weeks between the two study groups. Chi‐square tests were used to compare CSF sterilisation within 2, 4 and 12 weeks. Chi‐square and Fisher's exact tests were used to estimate the incidence of adverse events between groups. Statistical analyses were performed using SPSS statistics version 19 (IBM). All analyses were two‐sided and *P*‐values of < 0.05 were considered statistically significant.

## 3 RESULTS

### 3.1 Basic clinical characteristics

A total of 132 patients were excluded because of exclusion criteria including 50 patients present with severe hepatic or renal damage, 43 patients had accepted antifungal therapy administration before admission, 22 patients had prior surgical intervention due to intracranial hypertension and 17 patients with recurrent CM. There were 43 patients in group I treated with AmB and 5‐FC, whereas 47 patients in group II were treated with FCZ and 5‐FC. There was no clinical evidence of enrolled patients having occult underlying immune deficit based on patients’ past medical histories. All patients were *C. neoformans s.l*.‐positive. The demographics and baseline CSF parameters of the study patients are listed in Table 1. Patients in the FCZ and 5‐FC group had higher median CSF burden of cryptococcal organisms before treatment compared to patients in the AmB and 5‐FC group (median 3632 vs 900 Cryptococci/mL, *P* = 0.008). No significant differences were observed between groups with respect to age, gender, CSF opening pressure, CSF WBC, CSF protein and CSF glucose.

**Table 1:** Patient baseline characteristics

	AmB + 5‐FC (n = 43)	FCZ + 5‐FC (n = 47)	*P* value
Age, years (mean ± SD)	38.0 ± 14.5	43.9 ± 13.9	0.088
Gender, male (n, %)	27 (62.8%)	33 (70.2%)	0.507
CSF opening pressure mmCSF (med, range)	325 (80‐1070)	330 (130‐856)	0.685
CSF WBC count×10^6^/L (med, range)	50 (0‐340)	46 (0‐649)	0.753
CSF Protein g/L (med, range)	0.66 (0.05‐1.72)	0.78 (0.05‐4,51)	0.351
CSF glucose mmol/L (med, range)	1.88 (0.06‐3.56)	1.32 (0.03‐3.90)	0.056
CSF Cryptococci count/mL (med, range)	900 (1‐166929)	3632 (10‐120000)	0.008

Abbreviations: AmB, amphotericin B; 5‐FC, flucytosine; FCZ, fluconazole; med, median.

### 3.2 CSF sterility

CSF sterility results within 12 weeks are detailed in Table 2, and no significant differences were observed in the incidence of CSF cryptococcus clearance (32/43, 74.4% vs 33/47, 70.2%, *P* = 0.814) or time to clearance (39 days, range 20‐69 days vs 21 days, range 7‐63 days, *P* = 0.107) between group I and group II. There were no significant differences in early fungicidal activity (CSF sterility within 2 weeks) (1/43, 2.3% vs 4/47, 8.5%, *P* = 0.355) or persistent infection (CSF sterility beyond 4 weeks^14^) (24/43, 55.8% vs 19/47, 40.4%, *P* = 0.191) between group I and group II.

**Table 2:** CSF sterility data

	AmB + 5‐FC (n = 43)	FCZ + 5‐FC (n = 47)	*P* value
Case (n, %)	32 (74.4%)	33 (70.2%)	0.814
Days (med, range)	39 (20‐69)	21 (7‐63)	0.107
Cases ≤ 2 weeks (n, %)	1 (2.3%)	4 (8.5%)	0.355
>2 weeks (n, %)	31 (72.1%)	29 (61.7%)	
Cases, ≤ 4 weeks (n, %)	8 (18.6%)	14 (29.8%)	0.191
> 4 weeks (n, %)	24 (55.8%)	19 (40.4%)	

Note

Continuous variables analysed by t test; categorical variables analysed by chi‐square test or Fisher's exact test. Days indicate the time from diagnosis to the first negative CSF India ink stain.

Abbreviations: AmB, amphotericin B; 5‐FC, flucytosine; FCZ, fluconazole; med, median.

### 3.3 Treatment response

The primary treatment response outcome was evaluated in the 12th week following initial therapy. No significant difference was observed in the treatment response rate as shown in Table 3 (*P* = 0.402) between group I and group II. The complete response (26/43, 60.4% vs 23/47, 48.9%, *P* = 0.297), partial response (4/43, 9.3% vs 11/47, 23.4%, *P* = 0.093) and stable response (10/43, 23.3% vs 8/47, 17.0%, *P* = 0.599) were not significant differences between group I and group II. The total successful response rate (including both complete and partial responses) (30/43, 69.7% vs 34/47, 72.3%, *P* = 0.820) was not significantly different between group I and group II. Differences in incidence of disease progression (2/43, 4.7% vs 3/47, 6.4%, *P* = 1.000) and mortality (1/43, 2.3% vs 2/47, 4.3%, *P* = 1.000) were not significant between group I and group II.

**Table 3:** Treatment outcomes based on induction therapy

	AmB + 5‐FC (n = 43)	FCZ + 5‐FC (n = 47)	*P* value
Complete response (n, %)	26 (60.4%)	23 (48.9%)	0.297
Partial response (n, %)	4 (9.3%)	11 (23.4%)	0.093
Stable response (n, %)	10 (23.3%)	8 (17.0%)	0.599
Disease progression	2 (4.7%)	3 (6.4%)	1.000
Death	1 (2.3%)	2 (4.3%)	1.000

### 3.4 Adverse events

A total of 38 (88.4%) patients cannot tolerate recommended doses of AmB and 5‐FC for they needed dose reduction during the treatment attributed to adverse events. Details of all adverse events are provided in Table 4. The incidence of adverse events was lower in the FCZ and 5‐FC group compared to the AmB and 5‐FC group (9/47, 19.1% vs 39/43, 90.7%, *P* = 0.000). The most comment adverse event was hypokalaemia in both the AmB and 5‐FC group (39/43, 90.7%) as well as the FCZ and 5‐FC groups (8/47, 17.0%). The incidences of chills and fever (1/47, 2.1% versus 14/43, 32.6%, *P* < 0.001), phlebitis (1/47, 2.1% vs 8/43, 18.6%, *P* = 0.012), hypokalaemia (8/47, 17.0% vs 39/43, 90.7%, *P* = 0.000), gastrointestinal discomfort (1/47, 2.1% vs 8/43, 18.6%, *P* = 0.012) and liver impairment (8/47, 17.0% vs 20/43, 46.5%, *P* = 0.003) were lower in the FCZ and 5‐FC group compared to the AmB and 5‐FC group.

**Table 4:** Adverse events in patients with non‐HIV‐ and non‐transplant‐associated CM

	AmB + 5‐FC (n = 43)	FCZ + 5‐FC (n = 47)	*P* value
Adverse events (n, %)	39 (90.7%)	9 (19.1%)	0.000
Chills and fever (n, %)	14 (32.6%)	1 (2.1%)	0.000
Phlebitis (n, %)	8 (18.6%)	1 (2.1%)	0.012
Hypokalaemia	39 (90.7%)	8 (17.0%)	0.000
Gastrointestinal discomfort	8 (18.6%)	1 (2.1%)	0.012
Liver impairment	20 (46.5%)	8 (17.0%)	0.003
Renal impairment	8 (18.6%)	4 (8.5%)	0.218
Haematological impairment	10 (23.3%)	5 (10.6%)	0.157
Myocardial damage	2 (4.7%)	0	0.225

## 4 DISCUSSION

We found that the majority of patients with non‐HIV‐ and non‐transplant‐associated CM do not tolerate the recommended induction therapy doses of AmB or 5‐FC. Our findings were in accordance with those of previous studies which depict medication intolerance as a commonly documented problem in China.^15^, ^17^, ^18^ Although dosages of AmB and 5‐FC were adjusted, the incidence of adverse events in this study was still high (90.7%). AmB activity is concentration‐dependent, so it is vital to ensure that drug concentrations reach the level of therapeutic effect.^25^, ^26^ As a result of severe toxic side effects, CM patients are unable to endure the recommended dosages of AmB or 5‐FC, which may result in a corresponding decrease of fungicidal activity and response to treatment^14^, ^16^ even increase the risk of relapse.^20^, ^27^, ^28^, ^29^

FCZ is widely applied in clinical treatment of deep mycosis and high pharmacological efficacy with few adverse reactions. FCZ combined with 5‐FC has been recommended to treat HIV‐associated CM.^14^ However, limited comparative data related to FCZ combined with 5‐FC in treating non‐HIV‐ and non‐transplant‐associated CM exist. Total fungal infection burden is regarded as an important predictor of poor outcomes. In the present study, while the baseline fungal infection burden was higher in the patients treated with FCZ and 5‐FC, the total successful response rate, which include complete and partial responses, was higher compared to the AmB and 5‐FC treatment, although these differences did not reach significance (34/47, 72.3% versus 30/43, 69.7%; *P* = 0.820). Moreover, the incidence of CSF culture sterility at two weeks, indicative of early fungicidal activity, or at four weeks, indicative of persistent infection, was not significantly different between the groups. The rate of fungal clearance from the CSF can help us to evaluate the clinical outcomes of patients.^30^ These findings indicated that FCZ and 5‐FC was effective with satisfactory outcomes (72.3%) in treating non‐HIV‐ and non‐transplant‐associated CM.

Side effect rates during induction therapy in the FCZ and 5‐FC group were significantly lower than in the AmB and 5‐FC group (9/47, 19.1% vs 39/43, 90.7%, *P* = 0.000). The incidence of adverse events including chills, fever, phlebitis, hypokalaemia, gastrointestinal discomfort and liver impairment were significantly lower in the FCZ and 5‐FC group compared to the AmB and 5‐FC group. The most common adverse event detected in patients treated with FCZ and 5‐FC was hypokalaemia, and we suspect this might be attributed to the use of mannitol for dehydration in order to lower intracranial pressure. In fact, the incidence of adverse events caused by fluconazole was typically low with reports indicating that larger doses (1200 mg per day) do not increase toxicity.^31^

FCZ and 5‐FC has high pharmacological efficacy with few adverse reactions in treating non‐HIV‐ and non‐transplant‐associated CM. AmB dosages of 0.7‐1.0 mg/kg per day may promote better outcomes and less relapse compared to lower doses used in previous studies.^20^, ^27^, ^28^, ^29^, ^32^ However, AmB dosages are often decreased prematurely due to severe toxicity, a common problem we faced in our treatment of non‐HIV‐ and non‐transplant‐associated CM cases in China. Adverse reactions including thrombophlebitis, febrile reactions and anaemia as well as kidney, liver and cardiac toxicity may occur during treatment and slow treatment progression.^33^, ^34^, ^35^ Patients with CM who are older, those that have critical conditions, or combined diseases involving other organs such as liver and kidney may not be candidates for AmB treatment. An alternative drug escalation regimen for these specific CM cases was needed. FCZ and 5‐FC had acceptable successful response rates (72.3%) with low incidences of adverse events (19.1%). This combination was shown to be a good alternative treatment for non‐HIV‐ and non‐transplant‐associated CM in cases where patients were unable to tolerate the recommended AmB dosage or in those not suitable for AmB treatment.

Our study was limited due in part to its retrospective nature and the fact that the total number of enrolled patients was limited. Moreover, we did not further apply molecular methods to distinguish *C. neoformans s.l*. from *C. gattii s.l*. after CGB culture. As a result, a definite consensus regarding the efficacy of FCZ and 5‐FC therapy for non‐HIV‐ and non‐transplant‐associated CM was needed. Future studies, particularly additional prospective large‐scaled randomised controlled trials including molecular methods to distinguish cryptococcal species, are warranted in order to definitively determine the best standard of care in these patient populations.

In conclusion, our study found that the majority of patients with non‐HIV‐ and non‐transplant‐associated CM do not tolerate the recommended doses of AmB during induction therapy due to side effect severity. Adjusting the dosages and treatment regimen of AmB still resulted in a high incidence of adverse events in this group. We showed that FCZ and 5‐FC combination therapy had relatively high efficacy with few adverse events in treating non‐HIV‐ and non‐transplant‐associated CM. FCZ and 5‐FC combination treatment proved to be a good alternative therapy for patients with non‐HIV‐ and non‐transplant‐associated CM. This treatment regimen is particularly efficacious in patient groups that are unable to tolerate the recommended dosages of AmB or in those that are not suitable candidates for AmB treatment.

## CONFLICT OF INTEREST

There are no conflicts of interest to declare.
